# Accurate and Fast Thermal Sensing via Phase-Responsive Nanothermometers and Neural Networks

**DOI:** 10.1021/acs.nanolett.5c04787

**Published:** 2025-11-10

**Authors:** Marina París-Ogáyar, Liyan Ming, Fengchan Zhang, Erving Ximendes, Riccardo Marin, Ginés Lifante-Pedrola, Aida Serrano, Ana Espinosa, Patricia Haro-González, Jordi Hernando, Daniel Ruiz-Molina, Jaume Ramon Otaegui, Claudio Roscini, Daniel Jaque

**Affiliations:** † Nanomaterials for Bioimaging Group (nanoBIG), Departamento de Física de Materiales, Facultad de Ciencias, Universidad Autónoma de Madrid, 28049 Madrid, Spain; ‡ Nanomaterials for Bioimaging Group (nanoBIG), Instituto Ramón y Cajal de Investigación Sanitaria (IRYCIS), Hospital Ramón y Cajal, 28034 Madrid, Spain; § Instituto de Ciencia de Materiales Nicolás Cabrera, Universidad Autónoma de Madrid, Madrid 28049, Spain; ∥ Intelligent Optical Nanomaterials (IONs) Group, Department of Molecular Sciences and Nanosystems, Ca’ Foscari University of Venice, Via Torino 155/b, I-30170 Venice, Italy; ⊥ Institute for Advanced Research in Chemical Sciences (IAdChem), Universidad Autónoma de Madrid, 28049 Madrid, Spain; # Departamento de Física de Materiales, Facultad de Ciencias, Universidad Autónoma de Madrid, 28049 Madrid, Spain; ∇ Departamento de Electrocerámica, Instituto de Cerámica y Vidrio | CSIC, 28049 Madrid, Spain; ○ Instituto de Ciencia de Materiales de Madrid (ICMM-CSIC), 28049 Madrid, Spain; ◆ Departament de Química, Universitat Autònoma de Barcelona, Edifici C/n, Campus UAB, Cerdanyola del Vallès, Barcelona 08193, Spain; ¶ Catalan Institute of Nanoscience and Nanotechnology (ICN2), CSIC and BIST, Campus UAB Bellaterra, Barcelona 08193, Spain

**Keywords:** luminescence, nanoparticles, phase change materials, dye, lifetime, artificial neural networks

## Abstract

Accurate, rapid, and remote temperature sensing at the nanoscale is essential for applications ranging from monitoring cellular thermodynamics to thermal management of microelectronic devices. Luminescent nanothermometers are promising candidates; however, their deployment is hindered by limited thermal sensitivity and cross-sensitivity to environmental factors that mimic temperature-induced luminescence changes. We introduce fluorescent chromatic nanoswitchers (CNSs), comprising silica nanocapsules incorporating a fluorescent dye within a thermoresponsive matrix. The matrix undergoes a solid-to-liquid phase transition, yielding an exceptional fluorescence lifetime sensitivity of 19% °C^–1^ at 37 °C. Crucially, the lifetime-based thermal readout provided by CNSs is resistant to environmental interference, ensuring reliable, reproducible temperature measurements. To enhance CNS performance, we integrate artificial neural networks (ANNs) for advanced lifetime signal processing, enabling faster and robust thermal readouts. Proof-of-concept experiments show that the synergy between high-sensitivity lifetime-based nanothermometers and ANN-driven analysis paves the way for next-generation thermal sensing technologies, offering improved responsiveness, real-time capabilities, and enhanced accuracy.

## Graphical abstract

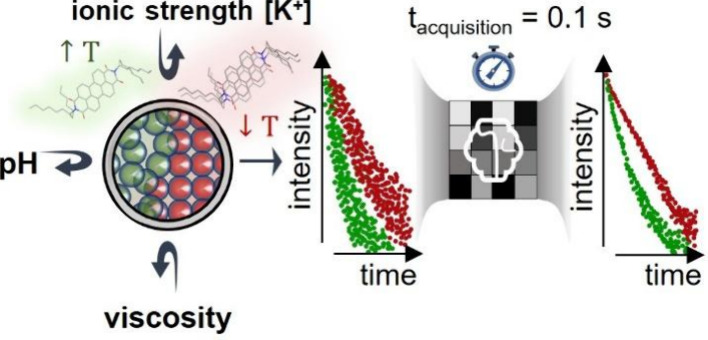

Temperature measurement has historically been a key concern given the crucial role of this parameter in numerous fields, including material fabrication, biology, diagnosis, environmental sciences, electronics, and food processing. Over the centuries, increasingly precise and accessible thermometric tools have been developed, from mercury thermometers to infrared thermal cameras. However, recent advancements in nanoelectronics, nanophotonics, and nanomedicine have rendered these traditional methods obsolete.^1^ For many applications, alternative thermal measurement technologies are required to provide thermal imaging with submicrometric spatial resolution and subdegree thermal precision.^2^ Additionally, remote data acquisition is highly desirable—particularly in biomedical applications, which require minimal disruption of cellular environments.^3,4^ Lastly, thermal sensors should be resistant against cross-sensitivity, i.e., they should provide thermal readouts despite changes in other physicochemical conditions such as pH, viscosity, magnetic fields, ionic strength, and humidity. A sensor immune from the effects of cross-sensitivity would, therefore, ensure reliable thermal measurements even in complex environments.^5^

Luminescent nanothermometers (LNThs) have emerged as a promising approach for remote temperature sensing.^6–8^ These nanoparticles or molecular probes exhibit luminescence that depends on temperature, allowing thermal readings with spatial resolution. In nanomedicine, the scientific community has identified the need to develop LNThs capable of achieving thermal and temporal precision of 0.1 °C and 0.1 s, respectively, particularly around physiological temperatures (37 °C).^9,10^ One strategy for achieving high thermal sensitivities in LNThs operating at 37 °C is the combination of luminescent units with thermosensitive materials that undergo structural phase transitions around this temperature.^11–19^ Among various studied systems, silica nanocapsules containing thermoresponsive luminescent solutions are particularly attractive. Indeed, recent findings demonstrate that the temperature-induced solid-to-liquid phase transition in these nanocapsules results in chromatic changes that remain independent of intracellular physiological parameters including pH, viscosity, and ionic strength.^15^ The thermoresponsiveness is granted by the core solution encased in the silica shell. The solution is composed of a luminescent dye – whose emission color depends on its aggregation state – and an hydrophobic phase change material with melting point of 37 °C – whose reversible solid-to-liquid phase transition modulates the solubility and thus the aggregation state of the dye. The spectral changes of CNSs have been leveraged for ratiometric thermal monitoring of processes characterized by small temperature changes such as intracellular heating due to metabolic activation.^15^ Despite these encouraging results, spectrum-based thermal readings generated by CNSs are limited in terms of achievable temporal and thermal resolution.

To improve measurement precision and temporal resolution in line with the goals set by the scientific community, several viable strategies have been explored. Among them, the use of fluorescence lifetime as a thermometric indicator and the implementation of artificial neural networks (ANNs) for advanced analysis of luminescent signals have shown particular promise.^20–29^ The combination of these strategies with a proven method to enhance reliability (i.e., CNSs) is expected to lead to a highly reliable luminescence thermometry approach. In this work, we systematically investigate the temporal dynamics of luminescence generated by CNSs to determine the optimal conditions for using fluorescence lifetime as a high-sensitivity thermal sensor at 37 °C. Additionally, we explore the potential of ANNs to simultaneously improve accuracy and precision of fast thermal measurements obtained from CNSs. The efficacy of this approach is validated through proof-of-concept experiments where CNSs are employed as thermal sensors under challenging measurement conditions.

## Temperature Response: Sensitivity and Reliability

The CNSs used in this study have an average diameter of 98 ± 22 nm (Figure 1a). The CNSs are composed of a commercial perylenediimide fluorophore (*N*,*N*′-bis­(1-hexylheptyl)­perylene-3,4:9,10-bis­(dicarboximide), PDI) embedded within a phase change material (PCM) matrix and enclosed in a silica (SiO_2_) shell (Figure 1b). The PCM is based on a mixture of the paraffins eicosane (EC) and docosane (DC) that shows a well-defined phase transition from solid to liquid near the physiological temperature range (*T* ∼ 37 °C).^15,30,31^ The thermofluorochromism of the CNSs is related to the photophysical properties of the PDI dye. The alkyl substituents at the *N*-imide positions enhance PDI’s solubility in liquid organic media displaying, in the monomeric state, a strong green emission accompanied by a short fluorescence lifetime (<10 ns, Figures 1c and 2a and Figure S1). Supramolecular aggregation of PDI molecules can occur under conditions such as increased concentration, unfavorable solute–solvent interactions, polarity effects, H-bonding, Coulombic interactions, or solid-state confinement. These processes typically involve π-π stacking, leading to the formation of aggregated excimer-like states with red-shifted emission and a slower decay component. In the present system, the PCM modulates the dye’s local environment, promoting aggregation and excimer emission in its solid state (in which the dye solubility is reduced), while restoring monomeric fluorescence once the mixture liquefies above its melting point (Figures 1c and 2a and Figure S1). The impact of phase transition on the fluorescence lifetime of PDI can be evaluated by defining an average lifetime value as

1 $$\tau_{av}\left(\lambda ,T\right)=\frac{\int I\left(\lambda ,t\right)t\;dt}{\int I\left(\lambda ,t\right)\;dt}$$

where *I*(*λ,t*) is the emitted intensity at wavelength λ obtained at time *t* after excitation. When the average lifetime is extracted from the decay curves using the eq 1, it renders τ_*av*_ (680 nm, 25 °C) = 25 ns and τ_*av*_ (520 nm, 50 °C) = 3 ns, revealing a remarkable difference in the fluorescence lifetime of PDI in the liquid and solid states of PCM.

**Figure 1:**
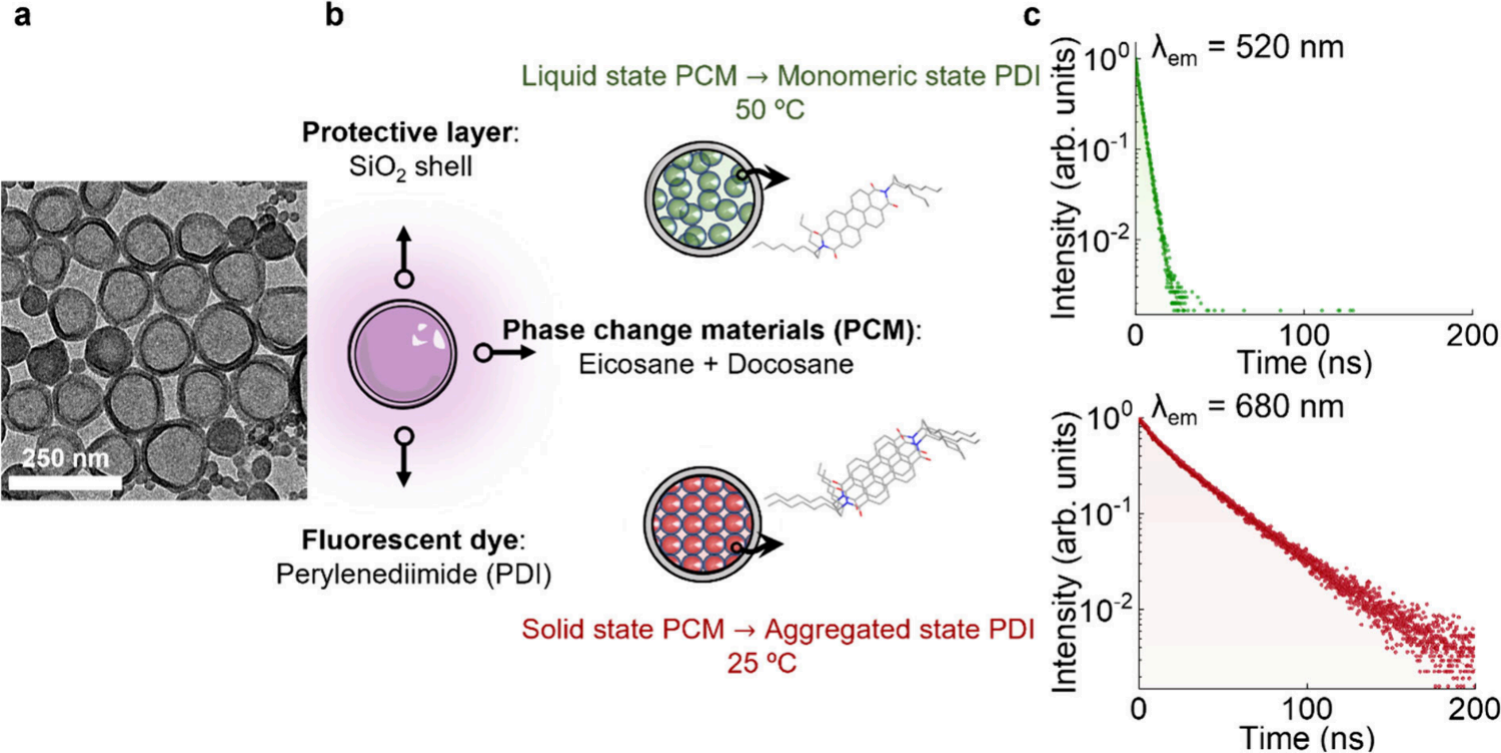
(a) Representative TEM image of the CNSs. (b) Schematic representation of the nanocapsules, composed of a protective silica layer loaded with a phase change material mixture (eicosane and docosane with a 1:1 weight ratio) and a fluorescent dye (perylendiimide, PDI). The phase change (from liquid to solid) induces the monomer–excimer emission of the dye. (c) Lifetime measurements of the monomer (green emission; λ_em_ = 520 nm) and excimer, i.e., aggregated (red emission; λ_em_ = 680 nm) state of the PDI. The excitation wavelength was λ_exc_ = 405 nm.

**Figure 2:**
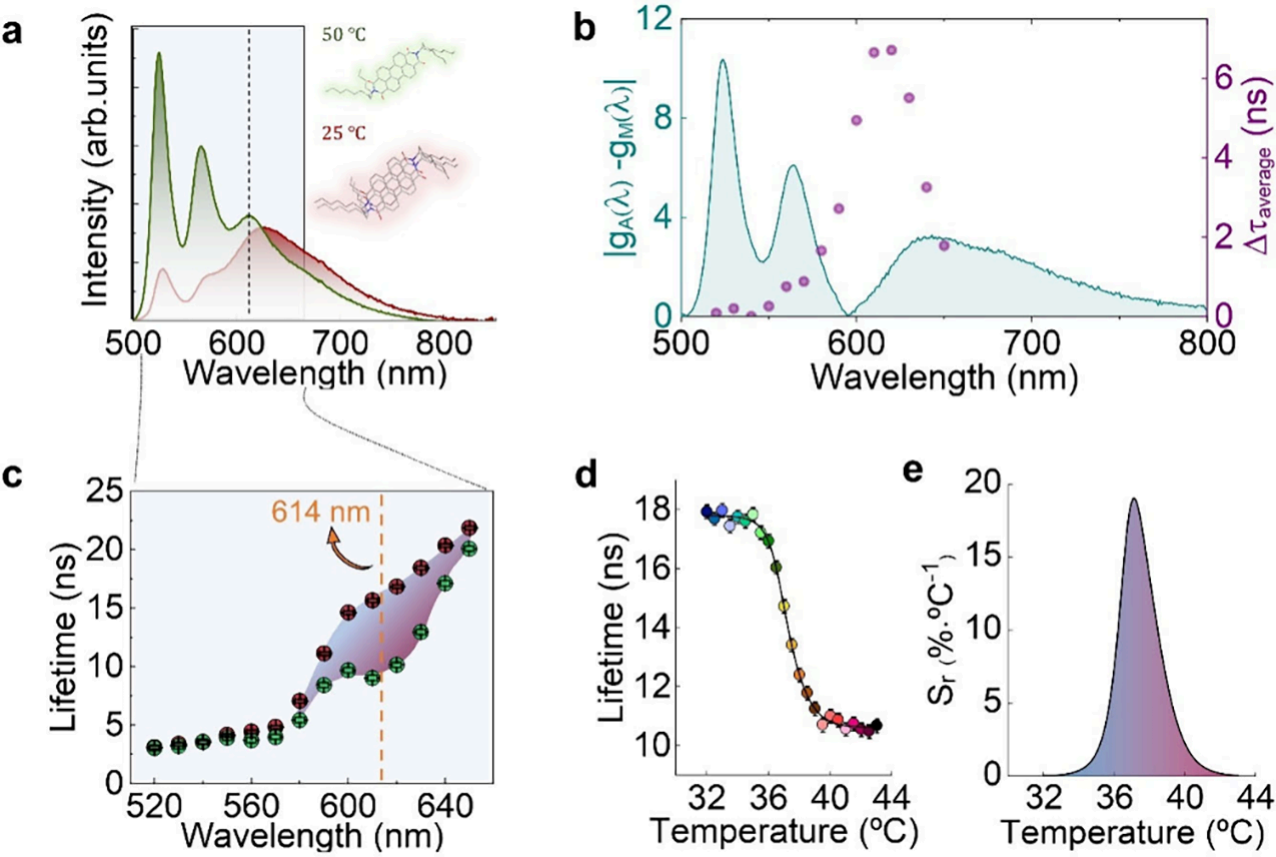
(a) Emission spectra of the monomer (50 °C, green emission) and excimer state (25 °C, red emission). (b) Comparison of the corresponding emission line shape functions in solid g_S_ (λ, 25 °C) and liquid g_L_ (λ, 50 °C) phases and wavelength dependence of the average lifetime difference, defined as Δτ_av_(λ) = τ_av_(λ, 25 °C) – τ_av_(λ, 50 °C). (c) Average lifetime values as a function of wavelength obtained at two different temperatures (25 and 50 °C, shown in red and green, respectively). The dashed line refers to 614 nm emission. (d and e) Average lifetime and sensitivity, respectively, as a function of temperature at λ_em_ = 614 nm. Symbols represent the measured average lifetime values every 0.5 °C, and the line is the corresponding fitting to a biphasic dose–response function. The relative sensitivity (*S*_r_) was calculated from the biphasic dose fitting. The excitation wavelength was set to λ_exc_ = 405 nm.

Decay curves included in Figure 1c correspond to PDI molecules in purely monomeric or excimer (aggregated) states. When the CNSs are at temperatures close to the phase transition the fluorescence decay curves of PDI molecules must be explained by adopting a two-population approach: the overall fluorescence signal results from the sum of the fluorescence generated by a population *N*_*A*_ of PDI molecules in the aggregated state (solid phase of PCM) and by a population *N*_*M*_ of PDI molecules in the monomeric state (liquid phase of PCM).^32,33^ Accordingly, the average fluorescence lifetime (τ_*av*_) would also depend on the relative contributions of the two molecular populations, and it could be described by

2 $$\tau_{av}\left(\lambda ,T\right)=\frac{I_{A}\tau_{A}+I_{M}\tau_{M}}{I_{A}+I_{M}}$$

*I*_*A*_ and *I*_*M*_ being the emitted intensities generated by the PDI molecules in the aggregated and monomeric states, τ_*A*_ is the fluorescence lifetime of PDI molecules in their aggregated state, and τ_*M*_ is their fluorescence lifetime in the monomer state. The population distribution between these two states is regulated by the temperature either promoting or inhibiting excimer formation. At the same time, the emission amplitudes of both the aggregated and monomeric states depend on the emission wavelength (Figure 1c), since each state emits primarily in a distinct spectral region—green for the monomer and red for the aggregated state. As a result, the average fluorescence lifetime of CNSs (PDI fluorescence) is expected to be both temperature- and wavelength-dependent. To anticipate the temperature and the emission wavelength that maximizes the thermal dependence of τ_*av*_ (maximizes the sensitivity of CNSs as lifetime-based sensors) we need to consider that the emitted intensities corresponding to the aggregated and monomeric states of the PDI molecules are given by

3 $$I_{A}\left(\lambda \right)=\alpha N_{A}g_{A}\left(\lambda \right);I_{M}\left(\lambda \right)=\alpha N_{M}g_{M}\left(\lambda \right)$$

where *g*_*A,M*_ (λ) are the emission line shape functions in aggregated and monomeric states, respectively, and α is a parameter that accounts for the emission quantum yield and for the optical coupling efficiency of the detection system in each of the states. In a first order estimation, α can be considered equal for both states. By including eq 3 in eq 2 and by differentiating with respect to temperature (see Section S2 of the Supporting Information), we obtain

4 $$\frac{d\tau }{dT}=\frac{dN_{A}\left(T\right)}{dT}\times \frac{\left[g_{A}\left(\lambda \right)\tau_{A}-g_{M}\left(\lambda \right)\tau_{M}\right]\times B\left(\lambda ,T\right)-\left[g_{A}\left(\lambda \right)-g_{M}\left(\lambda \right)\right]\times A\left(\lambda ,T\right)}{{\left[N_{A}\left(T\right)\left(g_{A}\left(\lambda \right)-g_{M}\left(\lambda \right)\right)+g_{M}\left(\lambda \right)\right]}^{2}}$$

with *B*(λ,*T*) and *A*(*λ,T*) being variables dependent on *N*_*A*_(*T*), *g*_*A,M*_(λ) and τ_*A,M*_. The maximum relative thermal sensitivity (S_r_) of the fluorescence lifetime, defined as *S*_*r*_ = τ^–1^ × *dτ*/*dT*, is expected when the term *dτ*/*dT* is maximized, which can be achieved in two ways according to eq 4. On the one hand, the derivative *dN*_*A*_(T)/*dT* reaches its maximum at the phase transition temperature, reflecting the sharpest change in the population of the states. On the other hand, one can minimize the denominator, i.e., achieve |*g*_*A*_(λ) – *g*_*M*_(λ)| = 0. This corresponds to the condition where the emission line shape functions of the two states are nearly identical. Figure 2b shows the wavelength dependence of |*g*_*A*_(λ) – *g*_*M*_(λ)|, from which we anticipate that maximum sensitivity would be achieved by recording decay curves for an emission wavelength close to 600 nm. To verify this prediction, we have systematically measured τ_*av*_ as a function of wavelength at 25 °C (aggregated state) and 50 °C (monomeric state). The results reveal how in the 520–590 nm spectral region, the average lifetimes at these two temperatures are nearly identical (∼ 5 ns) (Figure 2c). In this spectral range, luminescence arises primarily from monomeric PDI dye molecules so that *I*_*A*_ ≈ 0 and τ_*av*_ ≈ τ_*M*_ regardless of the temperature.^32,34–36^ For wavelengths larger than 640 nm, the emission from monomers is negligible (*I*_*M*_ ≈ 0) and the average fluorescence lifetime is that of aggregated state (τ_*av*_ ≈ τ_*A*_). As previously noted, this value remains unchanged at both 25 °C and 50 °C. In the 590–640 nm intermediate spectral region, highlighted in Figure 2c, a clear change in τ_*av*_ is observed when heating from 25 up to 50 °C. In this spectral region an overlap between the emissions corresponding to the aggregate and monomer states does exist (*I*_*A*_ ≠ 0 and *I*_*M*_ ≠ 0) and the average lifetime depends on the relative population of PDI dye molecules in their aggregate and monomer states. Experimental data in Figure 2c are in good agreement with our predictions based on eq 4: the maximum temperature modulation of fluorescence lifetime (τ_*av*_(20 °C) – τ_*av*_(50 °C)) takes place at ≈ 614 nm, very close to the wavelength at which |*g*_*A*_(λ) – *g*_*M*_(λ)| reaches its minimum value |*g*_*A*_(λ) – *g*_*M*_(λ)| ≈ 0 (Figure 2b).

Once the emission wavelength leading to the maximum temperature-induced modulation of τ_*av*_ was determined (614 nm), we systematically measured the τ_*av*_ vs *T* curve at this emission wavelength. The τ_*av*_ (614 nm, *T*) curve shows a sharp drop at the phase transition temperature from 18 to 10 ns corresponding to the gradual melting of the paraffines (Figure 2d). The lifetime-based *S*_*r*_ (Figure 2e) increases progressively near physiological temperatures, reaching a maximum sensitivity of 19% °C^–1^ at 37 °C. This sensitivity is larger than those reported for other lifetime-based luminescent nanothermometers (LNThs) that operate at physiological temperature, which generally show *S*_*r*_ values ranging between 1 and 5% °C^–1^ - albeit with few notable exceptions (Table S1 in Section S3).^3,12,17,37–46^ The τ_*av*_ vs *T* curves are also found to be robust against both changes in the physiological conditions of environments and repeated heating/cooling cycles (Figure 3). The lack of cross-sensitivity arises from the hydrophobic nature of the paraffins in which the dye molecules are dispersed, with the SiO_2_ shell providing additional structural support, mechanical robustness, and environmental isolation to the CNSs.^15,47–50^ The high thermal sensitivities, combined with the absence of cross-sensitivity and of temperature-induced damage, make CNSs an ideal system for precise and reliable lifetime-based thermal sensing, especially around 37 °C.

**Figure 3:**
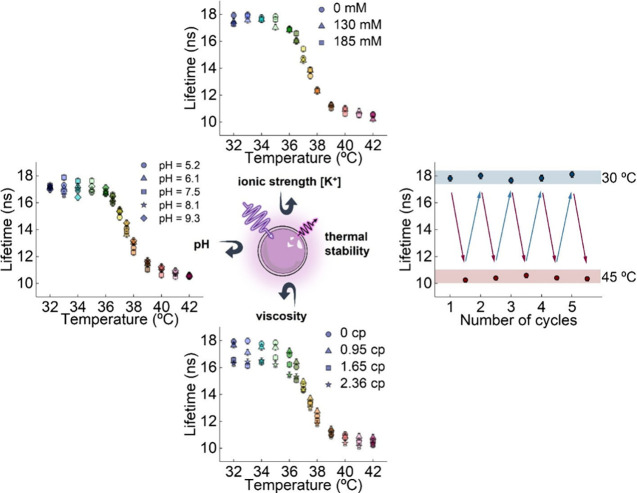
Assessment of the robustness of CNSs under physiological conditions, including ionic strength [K^+^], pH, and viscosity, as well as during repeated heating and cooling cycles. The excitation and emission wavelengths were set to λ_exc_ = 405 nm and λ_em_ = 614 nm, respectively.

## Improving Thermal Readout by Neuronal Networks

For a lifetime-based LNTh, the thermal resolution is given by δT = *S*_*r*_^–1^ × δτ/τ, where *δτ* is the experimental resolution in the measurement of the fluorescence lifetime.^2,10^ Precise determination of fluorescence lifetime traditionally relies on the acquisition of high signal-to-noise decay curves. The achievable values of signal-to-noise ratio depend on the brightness of the LNTh and on the acquisition time. For a given LNTh and detection system, reducing *δτ* thus requires extending the integration time (Figure S2), which compromises the temporal resolution (*δt*) of the thermal readout. The *δτ* for each integration time *δt* is experimentally evaluated by acquiring decay curves, applying eq 1 to raw data, and then performing statistical analysis on the τ_*av*_ values. When working with raw data and reducing the integration time to 0.1 s, *δτ* (raw) increases to 2.4 ns (Figure 4a, data obtained at 36.5 °C). Direct analysis of raw data (raw decay curves transformed to temperature values) leads to thermal resolutions of 2.9 °C when the integration time is reduced down to 0.1 s. These numbers are well above the δt δT = 0.01 °C s target.^10^ Furthermore, when the integration time is increased, the analysis of raw data leads to δt· δT values that differ even more from the 0.01 °C s target (Figure 4b). Therefore, it is evident that alternative approaches for the analysis of decay curves are required in order to approach the target value of 0.01 °C s.

**Figure 4:**
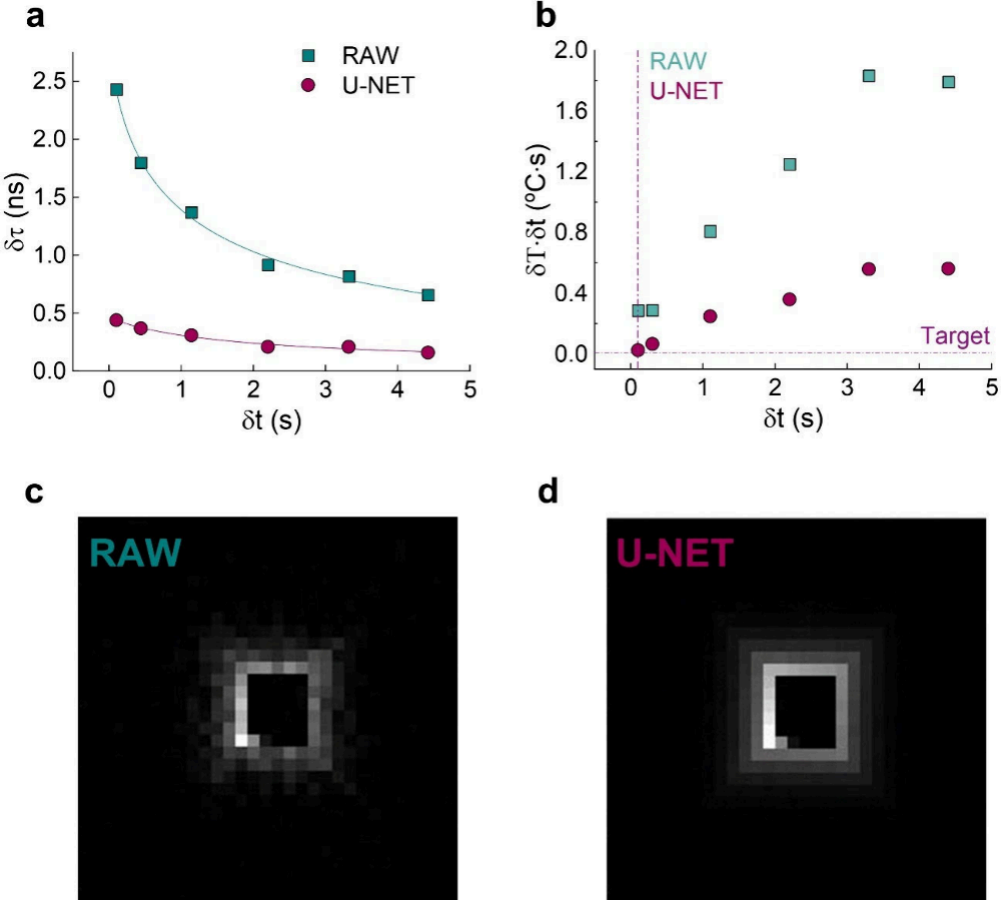
(a) Variation of the experimental resolution in the measurement of the fluorescence lifetime (*δτ*) with the acquisition time (*δt*), obtained from the analysis of raw decay curves. The results obtained after applying the U-NET to experimental data are also included. Decay curves were measured at 36.5 °C. Dots are experimental data, and lines are a guide for the eye. (b) Variation of product *δtδT* as a function of *δt* (time resolution, assumed to be the integration time of measurements) for raw data and after applying the U-NET. Dashed lines indicate the *δtδT* = 0.01 °C s target along with 0.1 s integration time. Data correspond to a temperature of 36.5 °C. (c) Grayscale square spiral image of a raw decay curve acquired with an integration time of 1.1 s at 36.5 °C (λ_exc_ = 405 nm, and λ_em_ = 614 nm). (d) Grayscale square spiral image of the decay curve included in panel c after applying the U-NET.

Deep learning techniques have proven to be effective in overcoming the effect of noise in the analysis of signals.^22–29^ U-shaped convolutional neural networks (U-NETs) are particularly suited to denoise measurements with Poisson errors. As such, they have been recently used to enhance the performance of lifetime-based Ag_2_S LNThs in low signal-to-noise ratio (SNR) conditions, increasing thermal readout precision without compromising temporal or spatial resolution.^24^ The approach consists in converting each decay curve into a gray scale image (by a spiral transformation) and use the trained U-NET to denoise the image. As such, a more precise readout of fluorescence lifetime is achieved (Figure 4c,d). To evaluate the suitability of this approach and to improve the performance of our CNSs, we first trained the U-NET. Detailed information on U-NET training and its procedure for generating denoised fluorescence lifetime values is provided in section S5. The U-NET output (Figure 4d) is a denoised image from which lifetime values are determined by multiplying the grayscale intensity of each pixel by the temporal bin width of the acquisition system. This is equivalent to applying eq 1. The *δτ* values provided by the U-NET obtained for different acquisition times (different *δt*) at 36.5 °C are more than 4 times lower than those provided by the analysis of raw data (Figure 4a). When *δτ* are converted into *δT*, it is evident how the use of U-NET algorithms makes possible to approach the δt· δT = 0.01 °C s target value (Figure 4b). It is worth highlighting that the improvements in *δτ* (and, consequently, in *δT*) provided by U-NET have not only been observed at 36.5 °C, but across the whole temperature range studied in this work (Figure S6). This increase in precision is also accompanied by an improvement in readout accuracy.

The actual potential of U-NET to achieve fast and precise thermal monitoring was finally validated through a proof-of-concept photothermal experiment (Figure 5a). The experimental setup consists of a thermoregulated chamber maintained at 36.5 °C, into which a 3 × 3 mm cuvette is inserted. The cuvette is illuminated with a 1450 nm laser (15 mW power) that acts as local heating source due to the strong water absorption at this wavelength. The cuvette contains 100 μL of water with a nanoparticle concentration of 2 mg/mL. Numerical simulations indicate that, in these experimental conditions, the 1450 nm laser should induce a fast increment in the average temperature of the cuvette from 36.5 up to 38.2 °C, with most of this increment being induced during the first seconds of heating (Figure 5b and Section S6). To monitor this heating process, the fluorescence decay curves (λ_*exc*_ = 405 nm and λ_*em*_ = 614 nm) of CNSs were acquired every 0.1 s (i.e., *δt* = 0.1 s) before and after switching ON the heating laser. Due to the reduced integration time, the decay curves recorded had low SNR values (Section S7). The time evolution of the average temperature is provided by applying the calibration curve (Figure S6b) to the time evolution of τ_*av*_ which was determined from the analysis of raw data as well as by applying the U-NET algorithm. The analysis of raw data reveals a starting temperature of 40 °C and a final temperature of 48 °C (Figure 5d,e). The starting temperature differs by more than 2.5 °C from the real (set point) starting temperature of the cuvette. This discrepancy reveals the limited reliability of the thermal data obtained from the analysis of raw data. In addition, the analysis of raw data provides a laser-induced heating as large as 8 °C, which is much higher than the average temperature anticipated by the numerical simulations (1.7 °C, Figure 5b and Figure S8). When the same decay curves are processed by the U-NET, a clear improvement in the thermal resolution is observed, decreasing down to 0.6 °C (Figure 5d). The U-NET- assisted analysis provides a starting temperature of 36.9 °C that differs by less than 0.5 °C from the set point temperature. This result highlights how U-NET-assisted analysis not only reduces thermal resolution but also increases the reliability of thermal measurements. The temperature vs time curve provided by U-NET can be fitted to an exponential rise function (solid line in the zoomed-in view of Figure 5c). The analysis yields a temperature increment of 1.3 °C and a characteristic heating time of 20 s, in very good agreement with numerical simulations (Figure 5b).

**Figure 5:**
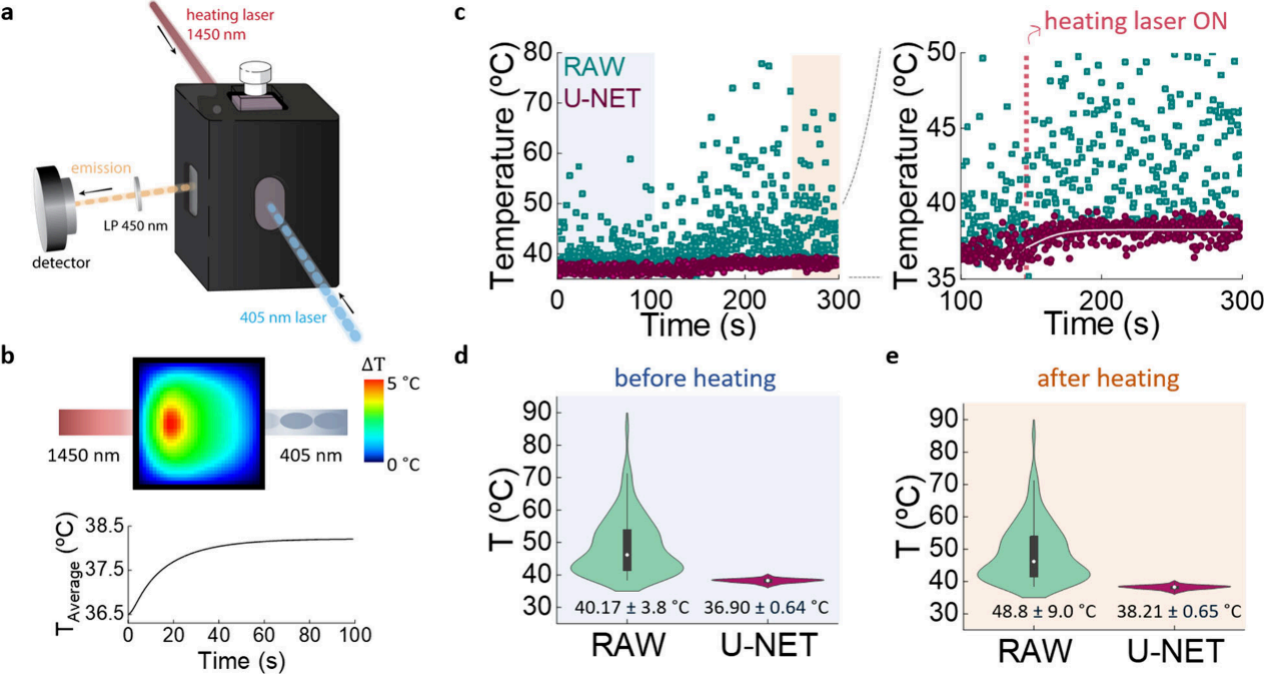
(a) Schematic representation of the proof-of-concept experiment designed to test the potential application of CNSs for fast and precise thermal monitoring. (b) Cross-sectional thermal image of the dispersion containing the CNSs within the cuvette when optically irradiated by the 1450 nm laser as provided by numerical calculation. The thermal image of the temperature increase (Δ*T*) has been calculated for a heating time of 100 s (steady state). The graph shows the time evolution of the average temperature of the dispersion within the cuvette. (c) Time evolution of the average temperature of the dispersion containing the CNSs within the cuvette as obtained from the analysis of raw data with the assistance of U-NET. The graph on the right is a close-up for better visualization. (d) Average and standard deviations (violin plots) of the temperature readouts obtained before starting the heating process (blue area in panel c). (e) Average and standard deviations (violin plots) of the temperature readouts obtained at the end of the heating process (light-red area in panel c, corresponding to the plateau temperature). Results from the analysis of raw and U-NET data are included for comparison.

In summary, we have demonstrated that reliable, precise, and rapid thermal sensing can be achieved by incorporating a commercial fluorescent dye within a phase change material (PCM) matrix enclosed in a silica shell, resulting in the formation of chromatic nanoswitchers (CNSs). The large differences between the fluorescence lifetime of dye molecules in the solid and liquid state of the PCM lead to a strong modulation of the average fluorescence lifetime of the CNSs. Experimental observations align closely with model predictions, both pointing to the spectral range and temperature where the temperature-induced lifetime variation reaches its maximum. For the optimum emission wavelength (614 nm) and near the phase transition temperature (36.5 °C), fluorescence lifetime has been found to decrease at rate of 19% °C^–1^ positioning this system among the most sensitive lifetime-based luminescent nanothermometers. The thermal sensitivity of the CNSs lifetime-based thermometry has been found to be independent of the physicochemical conditions of the surrounding medium, demonstrating the absence of cross-sensitivity and the CNSs robustness as thermal reporters. The potential application of the CNSs for accurate, precise and fast thermal sensing has been explored in a proof-of-concept experiment. We have found that, when thermal readout is obtained from raw data analysis, decreasing the integration times (time resolution) down to 0.1 s leads to an increment in the thermal resolution above 3 °C and to a limited reliability. This poor performance can be substantially improved by utilizing neuronal network algorithms for the analysis (denoising) of the experimentally obtained decay curves. Proof-of-concept experiments evidence how implementation of neural networks leads to a significant improvement in thermal resolution and readout accuracy. In addition to their use for laser-induced heating of solutions, we also investigated the potential of CNSs for real-time thermal monitoring in live cells. Unfortunately, our results revealed two major limitations that hinder reliable intracellular temperature sensing: the low internalization efficiency of CNSs into live cells, and the significant spectral overlap between CNS emission and cellular autofluorescence. As detailed in Section S8, these factors result in a mixed fluorescence signal that prevents accurate lifetime-based temperature measurements. To overcome these challenges, future designs of CNSs should incorporate near-infrared-emitting fluorescent units to minimize spectral interference, along with optimized functionalization strategies to enhance cellular uptake. These improvements will be essential for enabling precise and reproducible thermal sensing in complex biological environments.

The results included in this work pave the way toward the development and application of reliable, precise and fast luminescent nanothermometers by combining the design of new fluorescence nanomaterials showing strongly temperature dependent lifetimes with the application of algorithms for the advanced analysis and treatment of luminescence signals.

## Synthesis

The synthesis of chromatic nanoswitchers (CNSs) was carried out following the procedure described in ref 15. A hot (∼60 °C) aqueous solution containing 20 mL of poly­(vinyl alcohol) (2.5 mg/mL) was mixed with a solution of 6 mg of N,N’-bis­(1-hexylheptyl)­perylene-3,4:9,10-bis­(dicarboximide) dissolved in 50 mg of eicosane (EC) and 50 mg of docosane (DC), and 300 mg of methyltrimethoxysilane (MTMS) as the silica (SiO_2_) precursor.

An emulsion was formed using a Branson 450 W ultrasonicator equipped with a 1.2 cm sonication tip. To initiate hydrolysis and polycondensation of the SiO_2_ precursor, the pH was adjusted to 11 with aqueous ammonia (28%) and the mixture was left at 60 °C under gentle stirring for 16 h. Finally, the resulting CNSs were thoroughly washed 5 times through cross-filtration using a 500 kDa PES filter.

## Steady State and Luminescence Lifetime Measurements

Emission spectra and luminescence decay curves were recorded using an FS5 spectrofluorometer (Edinburgh Instruments), equipped with an SC-20 Temperature-Controlled Holder module for precise temperature regulation. Excitation was carried out using an EPL-405 laser (Edinburgh Instruments), and a long-pass filter (LP450) was placed before the detection system to eliminate laser scattering contributions. A consistent sample concentration of 2 mg/mL was used for all measurements.

To study the influence of different environmental parameters, we modified the sample conditions as follows: glycerol was added in varying amounts to adjust viscosity; hydrochloric acid and sodium hydroxide were used to modify the pH; and KCl was used to regulate the ionic strength [K^+^].

## Neural Networks

To train the U-shaped convolutional neural network (U-NET, as illustrated in Figure S4), we acquired decay curves across a range of SNR values by varying the integration time. Measurements were acquired at temperatures from 36 to 39 °C in steps of 0.5 °C. For each temperature, a long-integration acquisition (300 s) served as the high-fidelity ground-truth reference during training. Prior input to the network, we applied a spiral mapping to transform the temporal signal into a 2-D spatial representation that U-Net can exploit more effectively.^23^ Model optimization used the root-mean-square error (RMSE) as the loss function; the corresponding training and validation loss curves are presented in Section S5.

For the proof-of-concept experiment, a 1450 nm continuous-wave laser (P = 15 mW) was inserted into the SC-20 Temperature-Controlled Holder module, which was set to 36.5 °C. The laser was positioned on the side opposite to the excitation path of the 405 nm pulsed laser (EPL-405, Edinburgh Instruments), which was used to excite the sample within the cuvette.

All experiments presented in this study were performed using the same batch of nanoparticles, ensuring internal consistency across measurements. However, we acknowledge that batch-to-batch variability remains a challenge for future applications, and any new synthesis would require a dedicated calibration and retraining of the neural network to maintain accuracy.

## Simulations

The numerical simulations of the 3D maps of temperature of the dispersion containing the CNSs within the cuvette were obtained by solving the heat transfer equation.^51^ The heat term in the equation is provided by the energy deposited within the dispersion coming from the absorption of laser light at 1450 nm, where the absorption coefficient of the media at this wavelength is 3000 m^–1^. The heat transfer equation was numerically solved using a 3D-finite difference scheme based on the Douglas method, with appropriate boundary conditions at the cuvette walls.^52^ This numerical method assures a stable algorithm, and it is second order accurate in both space and time derivatives.

## Supplementary Material



## Notes

Additional experimental and computational details that complement the results presented in the main text, including fluorescence excitation spectra (Section S1), a model for predicting the optimal spectral range for measuring temperature (Section S2), benchmarking luminescent nanothermometers (Section S3), the impact of acquisition time on uncertainty (Section S4), U-NET training and operation (Section S5), heating simulations (Section S6), RAW decay curves (Section S7), and intracellular temperature sensing (Section S8) (PDF)
