# Supplementary material for: Accurate and Fast Thermal Sensing via Phase-Responsive Nanothermometers and Neural Networks
Source: Nano Lett. 2025 Nov 10;25(46):16538–46. doi: 10.1021/acs.nanolett.5c04787 (PMC12636073; doi:10.1021/acs.nanolett.5c04787)
Supplement: Supplementary file 1 [file nl5c04787_si_001.pdf]

Supporting Information for

# Accurate and Fast Thermal Sensing via Phase-Responsive Nanothermometers and Neural Networks

Marina París Ogáyar[1], Liyan Ming[1,2,3], Fengchan Zhang[1,3], Erving Ximendes[1,2], Riccardo Marin[1,4,5], Ginés Lifante-Pedrola[6], Aida Serrano[7], Ana Espinosa[8], Patricia Haro-González[1,3,5], Jordi Hernando[9], Daniel Ruiz-Molina[10], Jaume Ramon Otaegui[9,10], Claudio Roscini[10] and Daniel Jaque[1,2,5]*

[1]Nanomaterials for Bioimaging Group (nanoBIG), Departamento de Física de Materiales, Facultad de Ciencias, Universidad Autónoma de Madrid, 28049 Madrid, Spain.

[2]Nanomaterials for Bioimaging Group (nanoBIG), Instituto Ramón y Cajal de Investigación Sanitaria (IRYCIS), Hospital Ramón y Cajal, 28034 Madrid, Spain.

[3]Instituto de Ciencia de Materiales Nicolás Cabrera, Universidad Autónoma de Madrid, Madrid 28049, Spain.

[4]Intelligent Optical Nanomaterials (IONs) group, Department of Molecular Sciences and Nanosystems, Ca' Foscari University of Venice, Via Torino 155/b, I-30170 Venice, Italy.

[5]Institute for Advanced Research in Chemical Sciences (IAdChem), Universidad Autónoma de Madrid, 28049 Madrid, Spain.

[6]Departamento de Física de Materiales, Facultad de Ciencias, Universidad Autónoma de Madrid,28049 Madrid, Spain.

[7]Departamento de Electrocerámica, Instituto de Cerámica y Vidrio | CSIC, 28049 Madrid, Spain.

[8]Instituto de Ciencia de Materiales de Madrid (ICMM-CSIC), 28049 Madrid, Spain.

[9]Departament de Química, Universitat Autònoma de Barcelona, Edifici C/n, Campus UAB, Cerdanyola del Vallès, Barcelona 08193, Spain.

[10]Catalan Institute of Nanoscience and Nanotechnology (ICN2), CSIC and BIST, Campus UAB, Bellaterra, Barcelona 08193, Spain.

## Table of contents

**Section S1. Fluorescence excitation spectra (p. 2)**

**Section S2. Model to predict optimal spectral range for measuring temperature (p. 3)**

**Section S3. Benchmarking luminescent nanothermometers (pp. 4-5)**

**Section S4. Impact of acquisition time on uncertainty (p. 6)**

**Section S5. U-NET training and operation (pp. 7-8)**

**Section S6. Heating simulations (pp. 9-10)**

**Section S7. RAW decay curves (p. 11)**

**Section S8. Intracellular temperature sensing (p. 12)**

**References (pp. 13-14)**

## Section S1. Fluorescence excitation spectra

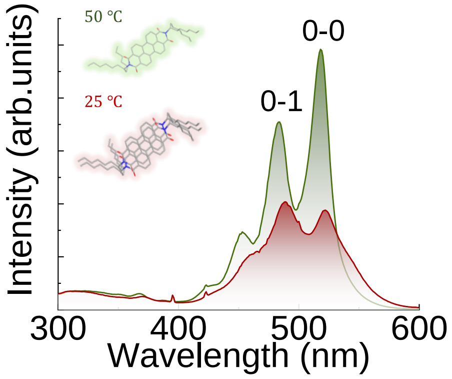


**Figure S1.** Fluorescence excitation spectra recorded for CNSs at two different temperatures. The peaks labeled 0-0 and 0-1 correspond to the vibronic transitions of the monomeric form of the dye. The emission wavelength was set to 614 nm.

## Section S2. Model to predict optimal spectral range for measuring temperature

The observed behavior can be modeled by expressing the total emitted light as a function of contributions from the solid and liquid states:

$$I_S(\lambda) = \alpha \cdot N_A \cdot g_A(\lambda)\ ;\ I_L(\lambda) = \alpha \cdot N_M \cdot g_M(\lambda)$$

where $N_{A,M}$ represent the population at each of the states ($N_A + N_M = 1$) and $g_{A,M}(\lambda)$ the corresponding emission line shape functions.

Equation of the lifetime as a function of the intensities:

$$\tau_{average} = \frac{1}{I_A + I_M}\,[I_A(\lambda,T) \cdot \tau_A + I_M(\lambda,T) \cdot \tau_M]$$

$$\tau_{average} = \frac{N_A(T) \cdot [g_A(\lambda) \cdot \tau_A - g_M(\lambda)\ \cdot \tau_M] + g_M(\lambda)\ \cdot \tau_M}{N_A(T) \cdot [g_A(\lambda) - g_M(\lambda)\ ] + g_M(\lambda)} \rightarrow \frac{A(T,\lambda)}{B(T,\lambda)}$$

Differentiating with respect to temperature, the following expression is obtained:

$$\frac{d\tau}{dT} = \frac{dN_A(T)}{dT} \cdot \frac{[g_A(\lambda) \cdot \tau_A - g_M(\lambda) \cdot \tau_M] \cdot B(\lambda,T) - [g_A(\lambda) - g_M(\lambda)] \cdot A(\lambda,T)}{\left[N_A(T) \cdot \left(g_A(\lambda) - g_M(\lambda)\right) + g_M(\lambda)\right]^2}$$

**Section S3. Benchmarking luminescent nanothermometers**

| Name | Material | Measurement type | Maximum relative sensitivity (%°C$^{-1}$) | Evaluated physiological parameters | Ref |
|---|---|---|---|---|---|
| NIPAM:TVPA = 200:1 | Nanogel composed of TVPA and NIPAM | Ratiometric | 128.42 (41 °C) in water | KCl (200 mM)<br>PH (5-8)<br>Both conditions modify the sensitivity by ratiometric sensing. | 1 |
| TICT@AIE | Aggregation-induced emission luminogen doped into a variety of natural saturated fatty acids | Lifetime Intensity | 17.72 lifetime<br>17.06 intensity<br>(37 °C) | KCl (500 mM)<br>PH (3-11)<br>PBS,RPMI +FBS<br>Normalized intensity at 590 nm was evaluated. | 2 |
| CdTe QDs@NaCl | CdTe Quantum Dots embedded in a NaCl Matrix | Lifetime | 52.8 (47 °C) | n.d. | 3 |
| Y@Gd:Nd (20%) | $NaYF_4$:Yb,Er@$NaGdF_4$:Nd nanoparticles | Ratiometric | ~9* (sensitivity show similar values over the temperature range of 30–50 °C) | n.d. | 4 |
| $CsPbCl_3$: 6% $Mn^{2+}$ PeQDs | $Mn^{2+}$-doped $CsPbCl_3$ perovskite QDs | Ratiometric | ~6* (30 °C) | n.d. | 5 |
| CdSe/$CdS_xSe_{1-x}$ MSQDs | CdSe/$CdS_xSe_{1-x}$ magic sized quantum dots | Ratio between the FWHM and the maximum emission intensity | 6.9 (32.85 °C) | n.d. | 6 |
| R-F127-MF NPs | Rhodamine dye-incorporated Pluronic F-127-melamine-formaldehyde composite polymer nanoparticles | Ratiometric | 4.9–15.4 in between 20–90 °C | PH(5-9)<br>NaCl(0-1000 mM)<br>Glycerol concentrations (20-80%) | 7 |
| UL-NC | Palladium(ii) 1,4,8,11,15,18,22,25-octabutoxypthalacyanine as the photosensitizer and 4-(5,6-dihydro-2-phenyl-1,4-oxathiin-3-yl)-N,N-dimethylbenzenamine as the photoenergy cache unit | Lifetime | 7.2 (30 °C) | n.d. | 8 |
| C-dots@OH | C-dots functionalized with–OH groups | Ratiometric | 5.6 (47 °C) | n.d. | 9 |
| TTA-Nd-NPs | Self-assembly of BDM & PtTPBP and β-NaYF4: 5%Nd | Ratiometric | 6.6* (30 °C) | PH (5-8) | 10 |
| ELP-TEMP | Elastin-like polypeptide fused with a cyan fluorescent protein, mTurquoise2, and a yellow FP, mVenus | Ratiometric | 45.1 (34 °C) | KCl (150 mM)<br>NaCl (150 mM)<br>PBS (EDTA 1mM)<br>$CaCl_2$ (1mM)<br>$MgCl_2$ (2mM)<br>PH (5-8). At 40 °C, the fluorescence ratio showed little variation within the pH range of 6–8, but at 45 °C and 50 °C it dropped when the pH fell below 6. | 11 |
| NR-Hy and NR-L64 | Nile Red-loaded hybrid (span 60-L64) niosomes and Nile Red-loaded L64 niosomes | Intensity<br>(some data for lifetime) | NR-Hy 19 (42 °C)<br>NR-L64 36 (40 °C) | PH (4-9)<br>NaCl (100-500mM). Slight variations in intensity at the highest concentrations of NaCl | 12 |
| R-CDs | Red-emissive CDs | Intensity | ≈13* (30 °C) | KCl (0-200 mM)<br>Several ions tested | 13 |

| | | | | | |
|---|---|---|---|---|---|
| CSS@Y | NaYF4:Yb/Er/Ce@NaYF4@NaYF4:Yb/Tm | Ratiometric | 9.86 (30 °C) | n.d. | 14 |
| Yb/Nd-NCs, $Ag_2S$ QDs HNM | Assembled $NaGdF_4$@$NaGdF_4$:Yb/Nd@Na$GdF_4$ + $Ag_2S$ into a nanocomposite material | Ratiometric | 7.8 (43.5 °C) | PH (5-9) | 15 |
| F1-POx and F2-Pox with γ-CD | Poly(2-oxazoline) polymer conjugated with tetraphenylenethene and hydroxyphenylbenzoxazole accommodated within the cavity of γ-cyclodextrin | Ratiometric | 274 (37.8 °C)<br>28.6 (33.2 °C) | n.d. | 16 |
| AIE/ACQ@PVA NPs | Saturated fatty acids wth Nile red and TPE-Al luminogens | Ratiometric | 63.66 (37 °C) | KCl (0-500 mM)<br>PH (3-11) | 17 |
| CNSs | Silica layer loaded with a phase change material (eicosane:docosane,1:1) and perylendiimide fluorescent dye | Lifetime | 19 (37 °C) | KCl (0-185 mM)<br>PH (5-9)<br>Viscosity (0-2.36 cp) | This work |

**Table S1. Selected literature reports** (> 5 % °C$^{-1}$, 30–50 °C, past 10 years).[1-17]
Variations observed under different physiological parameters are noted.
*Values marked with an asterisk were calculated by us from the information provided in the corresponding graphs.*

*For some works, the original temperature range reported extends beyond the range considered in this work.*

## Section S4. Impact of acquisition time on uncertainty

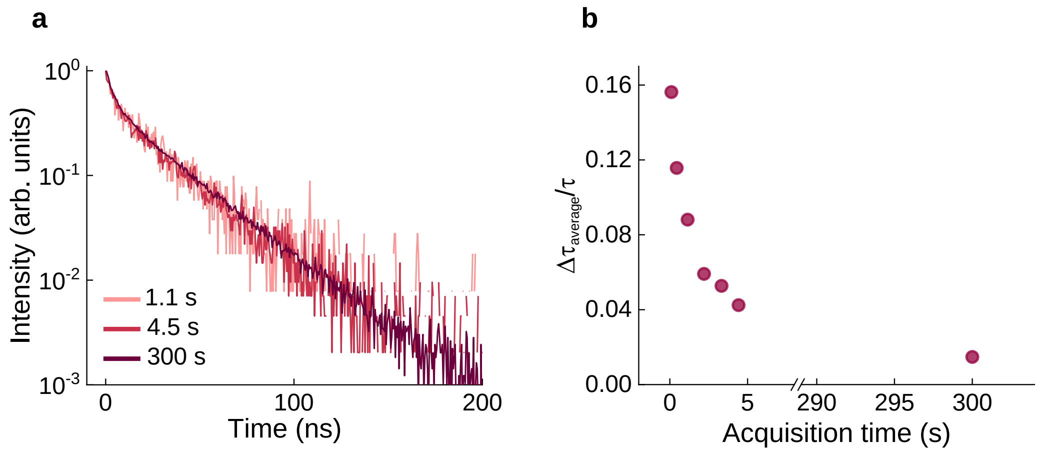


**Figure S2. a)** Representative fluorescence decay curves recorded with varying signal-to-noise ratios by adjusting the acquisition times. **b)** Relative uncertainty in the extracted average lifetime, expressed as $\Delta\tau_{average}/\tau$, plotted as a function of acquisition time. Each data point in b) was obtained by averaging statistical results from 50 individual curves.

## Section S5. U-NET training and operation

Training procedure consists, first, on setting the ground truth by recording, for temperatures between 36 and 39 °C, a decay curve with long acquisition times (300 s to ensure high signal-to-noise ratio (SNR), **Figure S3**). In a second step we trained the system with a total of 350 noisy curves, with different SNR, at different temperatures. Each decay curve was transformed to a greyscale sum (GSS) square spiral image (**Figure S4a**) which was provided to the U-NET algorithm.[18] The structure of the U-NET model shown in **Figure S4b.**

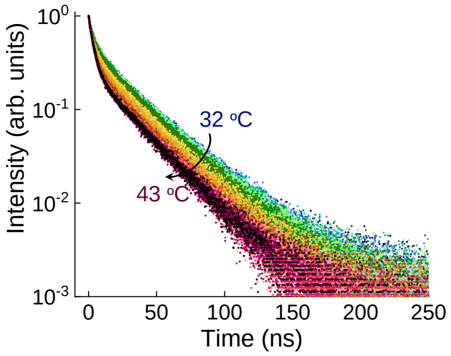


**Figure S3.** Fluorescence decay curves as a function of temperature. The excitation wavelength was $\lambda_{exc}$ = 405 nm, and the emission was collected at $\lambda_{em}$ = 614 nm. The acquisition time for each decay curve was 300 seconds.

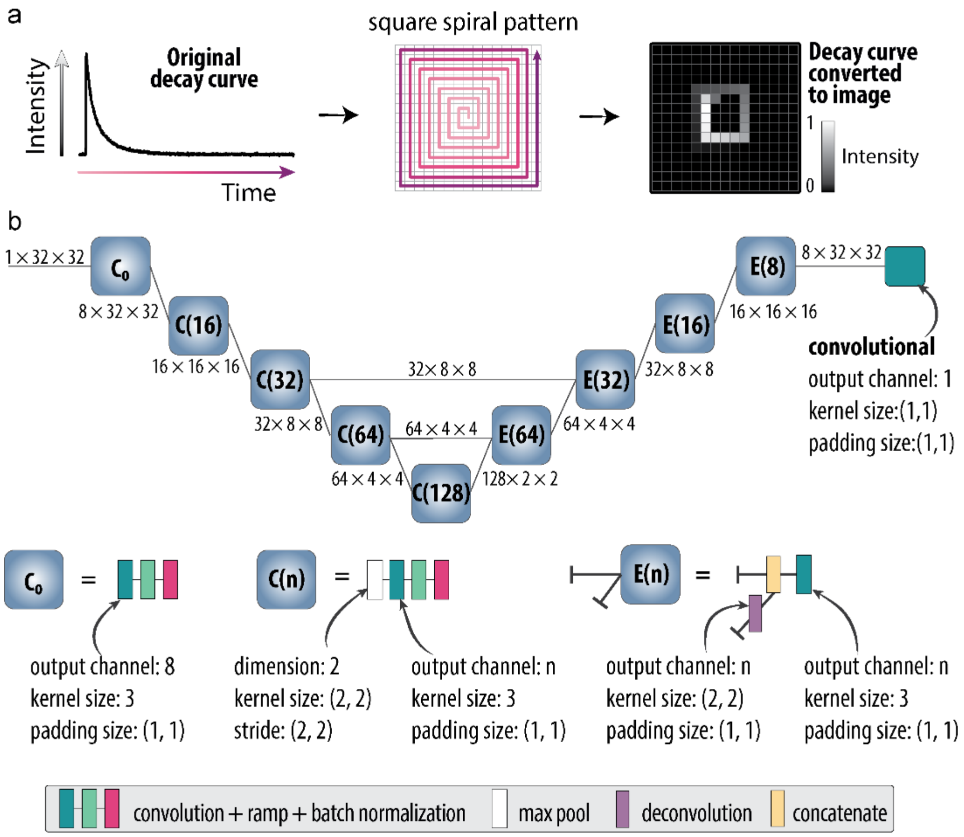


**Figure S4. a)** Conversion of decay curves into images by applying a square spiral mapping. **b)** Structure and hyperparameters of the utilized U-NET. Figures are adopted from previous work (doi.org/10.1002/adma.202306606).

During training, the loss—measured as root-mean-square error (RMSE)—decreased steadily across epochs, indicating that the U-Net progressively captured the salient structure of the luminescence decay curves. The validation RMSE followed a similar trajectory (**Figure S5**), suggesting that the learned representations generalize beyond the training set without evident overfitting.

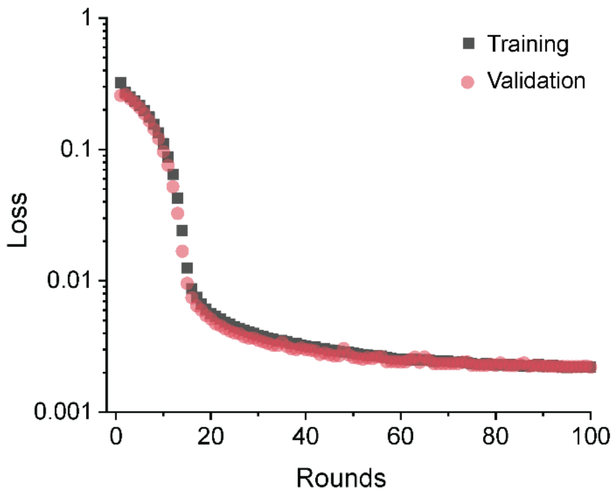


**Figure S5.** Learning curve of U-NET. The learning process seemed to be stabilized around the 100th round (approximately 60 min using the method of ADAM-Optimizer, batch size 64 and CPU).

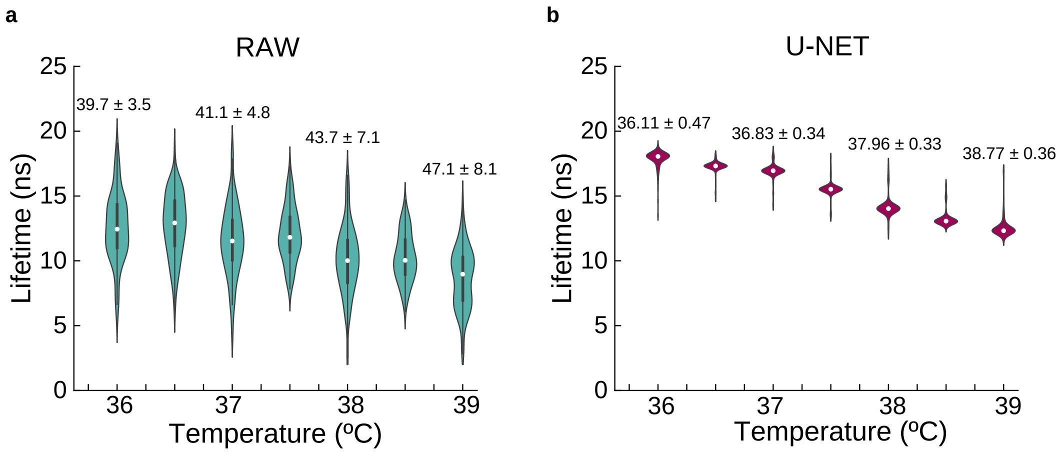


**Figure S6.** Calibration curves obtained from **a)** raw decay curves (with acquisition time 0.1 s) and **b)** after applying the U-NET model. The white dot indicates the median, the box represents the interquartile range and the whiskers extend to 1.5 times the interquartile range. The density plot is estimated using a kernel density function in Origin. The values plotted at the top correspond to the estimated temperature and its associated uncertainty in °C, while the x-axis represents the actual temperature set in the system, ranging from 36 to 39 °C in 0.5 °C increments.

**Section S6. Heating simulations**

Time evolution of the average temperature of the dispersion within the cuvette showed in **Figure 5b** in main text is calculated taking into account the attenuation of the 405 nm pulsed laser across the cuvette.

$$I = I_0 e^{-\alpha l} \qquad [\mathbf{1}]$$

$$O.D = log_{10} \frac{I_0}{I} \qquad [\mathbf{2}]$$

$$\alpha = \frac{O.D_{405\ nm}}{log_{10}e \cdot l} \approx 0.6$$

with an optical density at 405 nm ($O.D_{405nm}$) of 0.8, as obtained from **Figure S7**, and a cuvette path length of 3 mm (l = 3 mm).

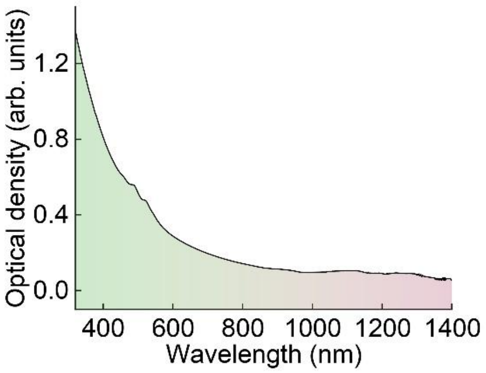


**Figure S7.** Optical density of a sample containing 100 µL of an aqueous suspension of CNSs (2 mg/mL) in a 3 × 3 mm cuvette.

The average temperature can be expressed by Equation **[3]**.

$$T_{Average} = \int_0^3 T(z) \cdot e^{-\alpha(3-z)} dz \qquad [\mathbf{3}]$$

By approximating **[3]** using Simpson's rule (with the values shown in **Figure S8c**), we obtained an average temperature increase of 1.7 °C.

$$T_{Average} \approx \int_a^b f(x)\, dx \approx \frac{h}{3}[f(x_0) + 4f(x_1) + 2f(x_2) + \cdots 2f(x_{n-2}) + 4f(x_{n-1}) + f(x_n)] \approx 1.7\ °C$$

where h is the step size between successive z-values ($h = \frac{b-a}{n}$).

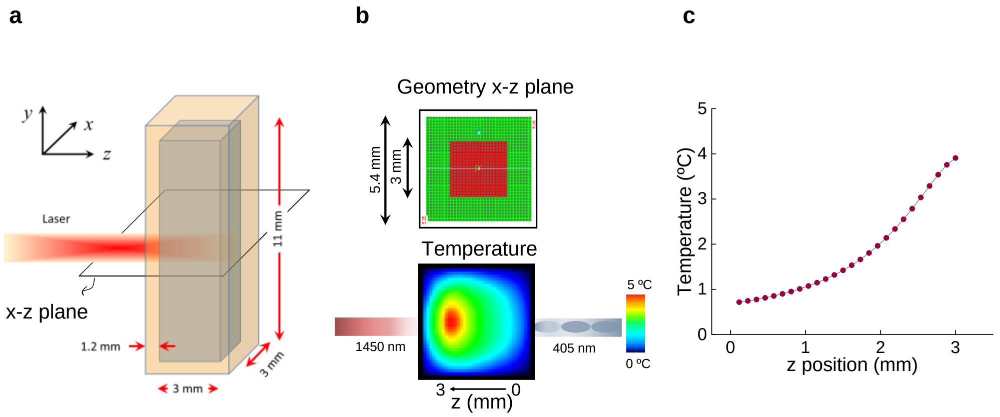


**Figure S8.** Heating simulations. **a)** Schematic representation of the cuvette dimension. **b)** Thermal distribution across the cuvette after 100 seconds of heating. The continuous irradiation was performed using a laser operating at 1450 nm with a power of 12 mW. The 405 nm pulsed laser is directed at the opposite face of the cuvette. **c)** Simulated temperature rise along the z-axis, corresponding to the thermal distribution shown in b).

## Section S7. RAW decay curves

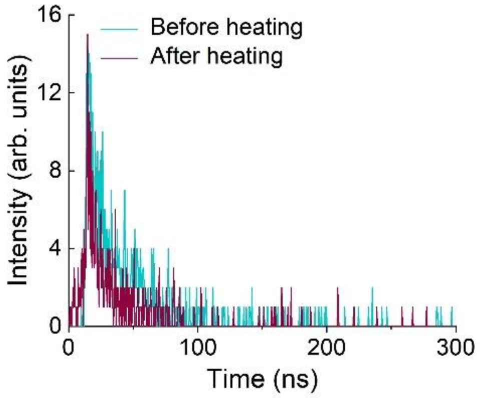


**Figure S9.** Example of the first and last raw decay curves (before and after heating, respectively) from the experiment shown in **Figure 5** of the main text.

## Section S8. Intracellular temperature sensing

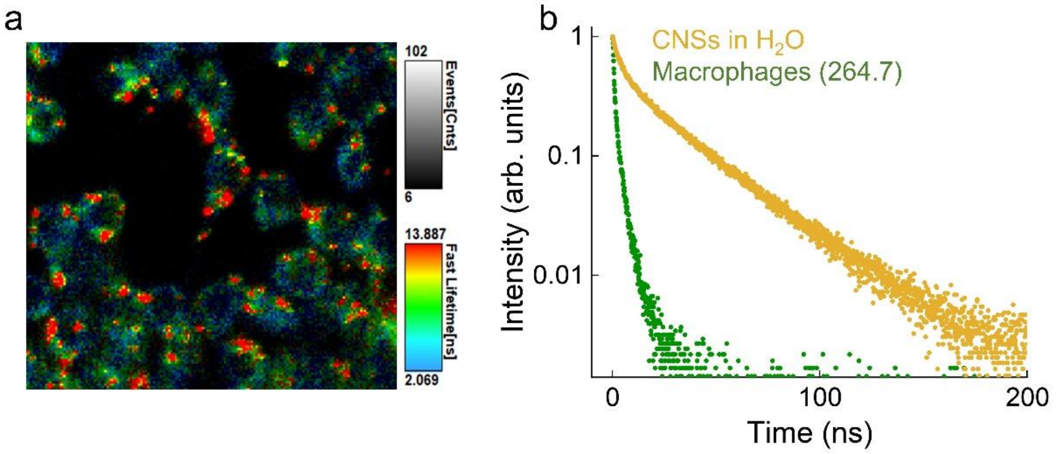


**Figure S10**. **a)** Fluorescence lifetime imaging (FLIM) of chromatic nanoswitchers inside RAW 264.7 macrophages. Image obtained at a temperature of 25 °C. The longest lifetime contributions (red) corresponding to the CNSs. The shorter lifetimes (blue–green) corresponding to the cellular autofluorescence. **b)** Decay curves corresponding to the autofluorescence signal generated by RAW 264.7 macrophage cells and that obtained in same conditions (25 °C, λexc= 405 nm, λem= 614 nm) for CNSs in an aqueous solution.

**Figure S10a** presents a representative fluorescence lifetime image of macrophages incubated with CNSs (cells are imaged at a temperature of 25 °C). The macrophages appear predominantly in blue-green tones, corresponding to lifetime values around 7 ns. This signal is tentatively attributed to the autofluorescence of live cells, which is known to exhibit short lifetimes. In contrast, the red spots in **Figure S10a** indicate regions with longer lifetimes (~14 ns), which we associate with the presence of CNSs.

However, this observed lifetime does not match the expected value for CNSs at 25 °C (see **Figure 3** in the main text). The discrepancy between the lifetime values obtained in the solution and in in vitro conditions (with the FLIM system) obeys to two different causes: the low internalization efficiency of CNSs into macrophages leads to low signal levels and, secondly, to the spectral overlap between the emission of CNSs and the broad autofluorescence band of the cells. This overlap results in a mixed signal, where the measured lifetime is an average of the short-lived autofluorescence (**Figure S10b**) and the longer-lived CNS emission. Due to these two reasons, it is not possible to isolate the CNS fluorescence from the cellular autofluorescence, thereby preventing the reliable use of CNSs as intracellular thermometers.

## References

1. Wang, C.; Zhao, X.; Wu, K.; Lv, S.; Zhu, C., A Ratiometric Organic Fluorescent Nanogel Thermometer for Highly Sensitive Temperature Sensing. *Biosensors* **2022,** *12* (9), 702.
2. Xue, K.; Wang, C.; Wang, J.; Lv, S.; Hao, B.; Zhu, C.; Tang, B. Z., A Sensitive and Reliable Organic Fluorescent Nanothermometer for Noninvasive Temperature Sensing. *Journal of the American Chemical Society* **2021,** *143* (35), 14147-14157.
3. Kalytchuk, S.; Zhovtiuk, O.; Kershaw, S. V.; Zbořil, R.; Rogach, A. L., Temperature-Dependent Exciton and Trap-Related Photoluminescence of CdTe Quantum Dots Embedded in a NaCl Matrix: Implication in Thermometry. *Small* **2016,** *12* (4), 466-476.
4. Xu, F.; Ba, Z.; Zheng, Y.; Wang, Y.; Hu, M.; Xu, X.; Wang, J.; Zhang, Z., Rare-earth-doped optical nanothermometer in visible and near-infrared regions. *Journal of Materials Science* **2018,** *53* (21), 15107-15117.
5. Chang, Q.; Zhou, X.; Jiang, S.; Xiang, G.; Li, L.; Li, Y.; Jing, C.; Ling, F.; Wang, Y.; Xiao, P., Dual-mode luminescence temperature sensing performance of manganese (II) doped CsPbCl3 perovskite quantum dots. *Ceramics International* **2022,** *48* (22), 33645-33652.
6. Silva, J. F.; Maria de Oliveira, J.; Silva, W. F.; Costa Soares, A. C.; Rocha, U.; Oliveira Dantas, N.; Alves da Silva Filho, E.; Duzzioni, M.; Helmut Rulf Cofré, A.; Wagner de Castro, O.; Anhezini, L.; Christine Almeida Silva, A.; Jacinto, C., Supersensitive nanothermometer based on CdSe/CdSxSe1-x magic-sized quantum dots with in vivo low toxicity. *Chemical Engineering Science* **2022,** *264*, 118153.
7. Wu, Y.; Liu, J.; Ma, J.; Liu, Y.; Wang, Y.; Wu, D., Ratiometric Nanothermometer Based on Rhodamine Dye-Incorporated F127-Melamine-Formaldehyde Polymer Nanoparticle: Preparation, Characterization, Wide-Range Temperature Sensing, and Precise Intracellular Thermometry. *ACS Applied Materials & Interfaces* **2016,** *8* (23), 14396-14405.
8. Su, X.; Wen, Y.; Yuan, W.; Xu, M.; Liu, Q.; Huang, C.; Li, F., Lifetime-based nanothermometry in vivo with ultra-long-lived luminescence. *Chemical Communications* **2020,** *56* (73), 10694-10697.
9. Han, Y.; Liu, Y.; Zhao, H.; Vomiero, A.; Li, R., Highly efficient ratiometric nanothermometers based on colloidal carbon quantum dots. *Journal of Materials Chemistry B* **2021,** *9* (20), 4111-4119.
10. Xu, M.; Zou, X.; Su, Q.; Yuan, W.; Cao, C.; Wang, Q.; Zhu, X.; Feng, W.; Li, F., Ratiometric nanothermometer in vivo based on triplet sensitized upconversion. *Nature Communications* **2018,** *9* (1), 2698.
11. Vu, C. Q.; Fukushima, S.-i.; Wazawa, T.; Nagai, T., A highly-sensitive genetically encoded temperature indicator exploiting a temperature-responsive elastin-like polypeptide. *Scientific Reports* **2021,** *11* (1), 16519.
12. Lazarus, R.; Kothari, R.; Venuganti, V. V. K.; Nag, A., Intracellular Temperature Sensing with Remarkably High Relative Sensitivity Using Nile Red-Loaded Biocompatible Niosome. *ACS Appl Bio Mater* **2025,** *8* (4), 3028-3039.
13. Wang, Q.; Tang, Z.; Li, L.; Guo, J.; Jin, L.; Lu, J.; Huang, P.; Zhang, S.; Jiao, L., Highly efficient red-emitting carbon dots as a “turn-on” temperature probe in living cells. *Spectrochimica Acta Part A: Molecular and Biomolecular Spectroscopy* **2022,** *280*, 121538.
14. Liu, H.; Yan, L.; Huang, J.; An, Z.; Sheng, W.; Zhou, B., Ultrasensitive Thermochromic Upconversion in Core–Shell–Shell Nanoparticles for Nanothermometry and Anticounterfeiting. *The Journal of Physical Chemistry Letters* **2022,** *13* (10), 2306-2312.
15. Li, D.; Jia, M.; Jia, T.; Chen, G., Ultrasensitive NIR-II Ratiometric Nanothermometers for 3D In Vivo Thermal Imaging. *Advanced Materials* **2024,** *36* (11), 2309452.
16. Li, Y.; Yu, H.; Li, H.; Sun, S.; Yu, R.; Xu, Y., Highly Sensitive Temperature Sensing in Biological Region with Ratiometric Fluorescent Response. *Molecules* **2025,** *30* (5), 1121.

17. Xue, K.; Huang, S.; Wu, K.; Sun, Z.; Fu, H.; Wang, C.; Wang, C.; Zhu, C., Ultrasensitive Ratiometric Fluorescent Nanothermometer with Reverse Signal Changes for Intracellular Temperature Mapping. *Analytical Chemistry* **2024,** *96* (27), 11026-11035.
18. Ming, L.; Zabala-Gutierrez, I.; Rodríguez-Sevilla, P.; Retama, J. R.; Jaque, D.; Marin, R.; Ximendes, E., Neural Networks Push the Limits of Luminescence Lifetime Nanosensing. *Advanced Materials* **2023,** *35* (52), 2306606.
